# Analysis of Land Cover Changes in Afromontane Vegetation of Image Forest Reserve, Southern Highlands of Tanzania

**DOI:** 10.1155/2020/7402846

**Published:** 2020-05-15

**Authors:** Canisius John Kayombo, Henry Joseph Ndangalasi, Cosmas Mligo, Richard A. Giliba

**Affiliations:** ^1^Forestry Training Institute, Olmotonyi, P. O. Box 943, Arusha, Tanzania; ^2^Department of Botany, College of Natural and Applied Sciences, The University of Dar es Salaam, P. O. Box 35060, Dar es Salaam, Tanzania

## Abstract

An analysis of land cover changes (LCCs) was done in Image Forest Reserve (IFR) from August to October 2019. Free satellite images for 1990, 2004, and 2018 were downloaded from Landsat 5 (TM) and Landsat 8 (OLI) available through the USGS portal. Ground surveys were conducted using systematically set plots of 20 m × 40 m to identify the existing land cover types and human illegal activities. Geographical coordinates for each of these plots were recorded using handheld GPS. We witnessed the changes of land cover types in the three decades. Forest had contracted, while shrubland and grassland and woodland had expanded within IFR. Between 1990 and 2004, woodland, bareland and rocky outcrops, shrubland, and grassland had consistently decreased though at a different rate of change, while forest has increased between the same assessment periods. The period of 2004–2018 has shown a consistent increase at different rates in woodland, bareland and rocky outcrops, shrubland, and grassland at a different rate of change, while forest has decreased between the same assessment periods. Further study is needed, bylaws and laws should be implemented, participatory forest management should be encouraged, beekeeping and ecotourism should be introduced, provision of regular education to the community by the Tanzania Forest Service (TFS) should be encouraged, and awareness creation should be made.

## 1. Introduction

The contribution of protected areas in biodiversity conservation has been appreciated in a wider geographical area by a number of studies [1–9]. Also, plants are well known for their role as carbon dioxide sinks [10, 11]. IFR is among protected areas of the southern highlands in Tanzania that was gazetted as a catchment forest reserve in 1954, an effort to increase conservation of natural resources in Tanzania. IFR is a part of the Eastern Arc Mountain (EAM) region, characterized with complex species-rich vegetation types [12, 13]. The diverse communities within the EAM make it harbour more than 40% of endemic plant species [14–17] are part of the Eastern Afromontane Biodiversity Hotspot [18–20]. Most of these species are localized either within natural forest cover types, as well as in the intervening habitats such as rocky outcrops, heathland, montane grasslands, and wetlands [21]. Despite its ecological importance, the ecoregion has been degraded through human activities [15, 22–26], consequently resulted in the land cover change and thus loss of biodiversity [27]. The land cover change is associated with modification of the earth's terrestrial surface [28], and the natural forests are more vulnerable [29].

Tanzania lost approximately 10 million ha of forest cover between 1970 and 1998 through clearing of forests for agriculture and livestock [30], thus, drawing attention to both local and global communities on the importance of conserving biodiversity in the Eastern Arc Mountains' ecosystem. Globally, it is known that human activities largely contribute to the land cover changes [31] and the decline in quality and quantity of natural species habitat types [32]. Nearly three billion people worldwide primarily depend on forests as their main energy source [33], and these attachments contribute to the depletion of vegetation cover and loss of natural habitats. The tropical montane forests are vital as habitats for plant species and carbon storage [34, 35]. However, they have been negatively encroached by the surrounding local communities because of high demand for fuel wood, fibres grazing land, timber construction poles and land for cultivation [36, 37], and other services of spiritual values [11]. The increase in illegal human activities within these forests may have made endemic and threatened species reported to exist in these forests [38, 39] and be driven to extinction as their natural forest cover became reduced beyond the ability to sustain a large number of species [20].

IFR is among Afromontane forests in Tanzania whose land cover types are threatened by human activities, regardless of being gazetted as a protected area [40]. It has been reported that demand for forest resources has grown over the years because of the human population growth in the surrounding communities [41]. However, the extent of land cover changes following the influence of human activities within the forest remains less known. The intensities of drivers of land cover are paramount important to understand the natural forest cover changes at local scales as pointed out by Potapov et al. [42] and Douwers et al [43], because a broad view can accurately be described at local scales where an obvious change is quantifiable and verifiable. The development of GIS and remote-sensing technology in the early 1990s was a breakthrough in land cover analysis, and a number of studies have been conducted to classify land cover types and changes associated with losses in tropical forests [44] and the major drivers of the existing changes. Generally, the land cover types and changes have been established at regional and global scales [45] but have not widely quantified changes at the local scale. With exiting studies focusing on understanding the major drivers of land cover changes at different spatial and temporal scales [46, 47], it was imperative to extend these understanding to the EAM forests of which IFR was the potential candidate. It was thus intended to establish land cover type changes and the existing changes between 1990 and 2018 within IFR. The major assumptions were that human activities outweigh natural factors on the dynamics of land cover types over large time intervals, and the duration between 1990 and 2018 was ideal to analyze changes that have occurred within IFR.

## 2. Materials and Methods

### 2.1. The Description and Location of the Study Area

**Figure 1:**
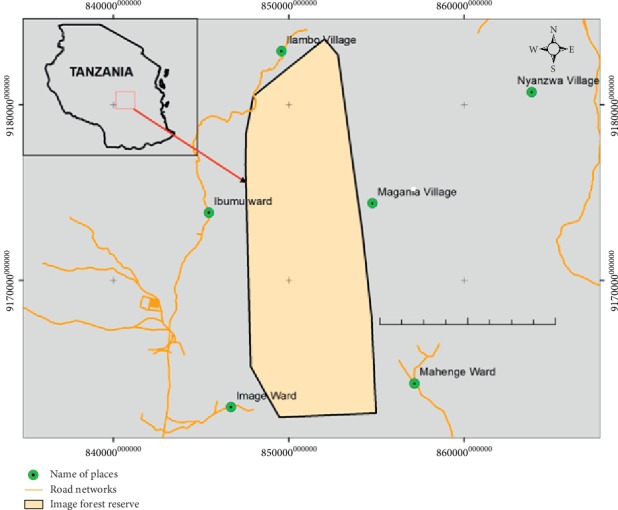
Map showing location of Image Forest Reserve (IFR).

IFR is found in Kilolo District, Iringa Region. The reserve falls under three wards, namely, the Image ward (to the south west), Ibumu ward (to the west and northwest), and Mahenge ward (to the east) (Figure 1). It is located at 07°22′15″–07°33′15″ South and 36°08′15″–36°12′25″ East (Figure 1). The IFR covers an area of 9,118.08 ha and was established as a catchment forest reserve in 1954. The landscape within IFR is characterized by rolling scenery and plateaus at an altitude range between 1640 m and 2440 m.a.s.l. [48]. IFR experiences oceanic rainfall with continental temperatures [49]. The area experiences one season of rain from November to April with an annual rainfall of 1500 mm [40], and the annual temperature ranges between 15° and 20°C [41]. IFR experiences oceanic rainfall with continental temperatures [49]. IFR experiences only one season of rain from November to April, with an annual rainfall of 1500 mm [40], and annual temperature ranges between 15°C and 20°C [41].

#### 2.1.1. The Vegetation Types Present in the Study Area

The plant communities within IFR are divided into natural forest, woodland, shrubland, and grassland that provide obvious physiognomic characteristics. The natural forest is dominated with the tallest trees, with height exceeding 35 m and the canopy cover reaching ≥95%. The dominant tree species in the natural forest included *Ilex mitis, Polyscias fulva, Craibia brevicaudata, Rapanea melanophloeos, Nuxia congesta, Dombeya torrida, Podocarpus latifolius, Hagenia abyssinica, Vepris simplicifolia*, and *Zanthoxylum deremense.* The woodland is dominated with *Brachystegia spiciformis, B. utilis, B. boehmii*, *Julbernardia globiflora,* and *Albizia antunesiana* mixed with *Uapaca kirkiana.* Shrubland is typical of miombo woodland species. The grassland is also well represented in areas without the closed canopy of woody plant species, making IFR have distinct patches either within the woodland or forest. The shrub and grass species with very scattered trees also extend in the areas with rocky outcrops' cropping landscapes that were dominated with *Hymenodictyon floribunda* and *Dissotis melleri*, *Myrothamnus flabellifolius*, and some plant species of sedges and grasses.

#### 2.1.2. The Population and the Socioeconomic Activities of Local Communities Surrounding Image Forest Reserve

IFR is surrounded by three wards (Ibumu, 6,681; Image, 9,180; and Mahenge, 10,039) making an overall population size of 25,900 [41]. The villages that are in close proximity with the IFR include Iyai and Kilalakidewa in the Image ward, Ibumu and Ilambo in the Ibumu ward, and Magana, Ilindi, and Nyanzwa in the Mahenge ward. The local communities adjacent to IFR practise both pastoralism and crop cultivation. Pastoralism involves a kind of free movement of livestock, and cultivation is mainly of mixed type that involves permanent and shifting cultivation. Irrigation farming is prominent during dry season and mostly practised very close to IFR's boundary, especially at the Iyai village in the Image ward, while for Ibumu and Mahenge wards, cultivation is done very close to the buffer zone. The livestock that are kept around the forest area include cows, goats, and donkeys which are essentially allowed to roam freely within the public land, but sometimes the local communities drive their livestock in IFR illegally, hence contributing to degradation of the forest reserve. Suitable climatic condition supports cultivation of maize (*Zea mays* L.), beans (*Phaseolus vulgaris* L.), and sugarcane (*Saccharum officinarum* L.) [41] has resulted into clearance of pristine land cover types for establishing new farms and consequently changes land cover to unforeseeable new cover types.

### 2.2. Data Collection

#### 2.2.1. Imageries and Ground Surveys

Free satellite images from Landsat 5 (TM) and Landsat 8 (OLI) available through the USGS (United States Geological Survey) portal were downloaded. The images selected were from dry season for the period 1995, 2005, and 2018 in order to acquire images with minimum cloud cover (<10%) and avoid differences due to seasons. Ground surveys were conducted from May to July 2019 to identify the existing land cover types in IFR and to develop classification scheme for land cover mapping. In each land cover type, the geographical coordinates were recorded using a handleheld GPS receiver. Human activities such as tree cutting, encroachment, livestock grazing, and wildfires, as drivers for land cover change, were recorded within the 170 set plots of 20 m × 40 m during the ground surveys.

*Human Illegal Activities*. During this study, any illegal activities observed outside the sample plots and not encountered before were also recorded during traversing within the plots and transects. The encountered tree stumps were counted and being assigned scores of 1, 2, 3, 4, and 5 for 1–10 countered stumps, 11–20 stumps, 21–30 stumps, 31–40 stumps, and ≥41 stumps, respectively. All other human activities, besides tree cutting, were estimated in form of percentage of damage occupancy on a plot of 20 m × 40 m and assigned scores of 1, 2, 3, 4, and 5 for 1–20%, 21–40%, 41–60%, 61–80%, and ≥81%, respectively.

### 2.3. Data Analysis

#### 2.3.1. Image Processing

Image processing involved three tasks, these include (i) image preprocessing, both visual and digital image processing was done, and prior to image processing, images were extracted from the full scenes to subset scenes into the area of interest which is IFR using ArcGIS 10.5 software. (ii) Image rectification were performed in order to ensure accurate identification of temporal changes and geometric compatibility with other sources of information, and the images were geocorded to the coordinate and mapping system of the national topographic maps, i.e., the UTM coordinate zone 36 south, Spheroid Clarke 1880, Datum Arc 1960. (iii) Atmospheric correction to convert digital numbers (DNs) to radiance based on the rescaling factors provided in the metadata files and convert radiance to the top of the reflectance (iv) Image enhancement, in order to reinforce the visual interpretability of images, a colour composite for Landsat 5 TM bands 4, 3, and 2 and Landsat 8 bands 5, 4, and 3 was prepared and its contrast was stretched using a histogram equalization to further enhance visual interpretability of linear features such as land-use features such as bareland and rocky outcrops. All image processing was carried out using ArcGIS software and Impact toolbox.

#### 2.3.2. Image Classification

Supervised image classification using the maximum likelihood classifier (MLC) was used to create land cover maps for the year 1990, 2004, and 2018. The maximum likelihood classifier was selected since, unlike other classifiers, it considers the spectral variation within each category and the overlap covering the different classes.

#### 2.3.3. Accuracy Assessment

Land cover maps derived from classification of images usually contain some sorts of errors due to several factors that range from classification techniques to methods of satellite data capture. Hence, evaluation of classification results is an important process in the classification procedure. Among the common measures to be used for measuring, the accuracy of thematic maps derived from multispectral imagery is an error/confusion matrix. An error matrix is a square assortment of numbers defined in rows and columns that represent the number of sample units assigned to a particular category relative to the actual category as confirmed on the ground.

#### 2.3.4. Land Cover Change Analysis

A cross-tabulation model was used to detect land cover change in ArcGIS, through which a land cover change matrix was produced. From a matrix, the change in land cover change was analyzed to depict gains and losses for the 1st period (1990–2004) and the 2nd period (2004–2018). Estimation for the rate of change for different land cover changes was computed based on the following formulae:

(1) $$\begin{array}{c} \%\text{land cover change}=\frac{\text{area of }i\text{th year }X+1}{\sum \text{area of }i\text{th year }X}\times 100\%,\\ \text{annual rate of change }=\frac{\text{area of }i\text{th year}+1}{t\text{th years}}, \end{array}$$

where area_*i* year *x*_ = area of cover *i* at the first date, area _*i* year *x* *+* *1*_ = area of cover *i* at the second date, ∑_*i*=1_^*n*^area_*i*year *x*_ = total cover area at the first, and *t*_years_ = period in years between the first and second scene acquisitions.

The ANOVA test was applied to test the significant change differences within the land cover types. The ANOVA test is used to determine the influence that independent variables have on the dependent variable in a regression study [50].

## 3. Results

### 3.1. Image Forest Reserve Land Cover Maps

**Figure 2:**
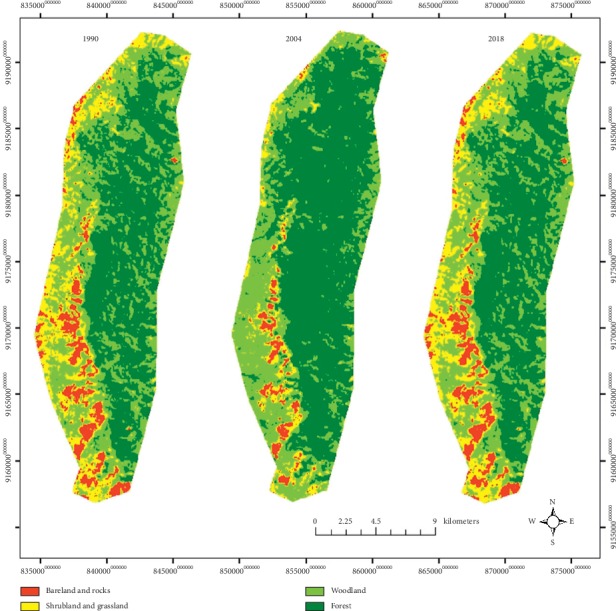
Land cover change maps of the IFR for the year 1990–2004 and 2004–2018.

**Figure 3:**
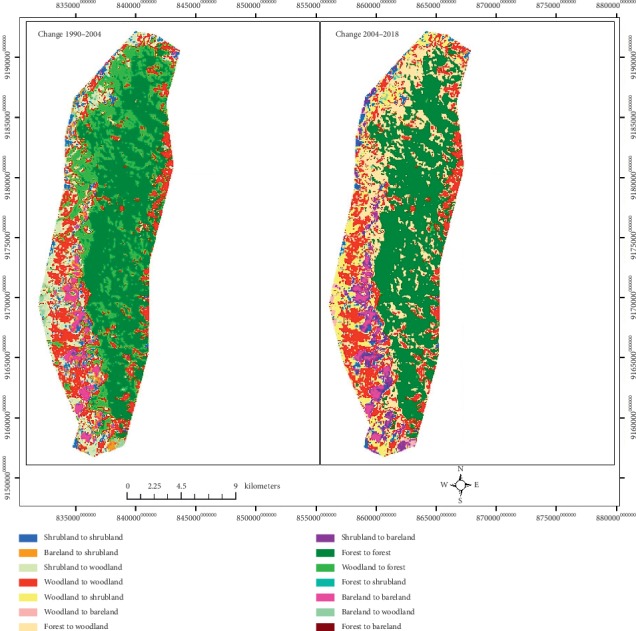
Land cover maps of the IFR for the year 1990, 2004, and 2018.

Over the past three decades, IFR has experienced changes in its land cover (Figure 2, Table 1). These changes have been exhibited throughout the IFR except in the eastern part. Forest has contracted, while shrubland and grassland and woodland have expanded within IFR (Figure 3).

Results for the first assessment period of 1990–2004 (Table 1) have shown two directional changes of the land cover changes: (i) woodland, bareland and rocky outcrops, shrubland, and grassland have consistently decreased though at a different rate of changes and (ii) forest has increased. Similarly, results for the second analysis period of 2004–2018 (Table 1) have shown two directional changes of the land cover changes: (i) woodland, bareland and rocky outcrops, shrubland, and grassland have consistently increased though at a different rate of changes and (ii) forest has decreased. Forest and woodland remained the dominant land cover representing a range of more than 80% to 90% of the entire land cover since 1990 to date (Table 1). However, the trends of their land cover change in the first assessment period have shown opposite direction with forest increasing and woodland decreasing. In the second assessment period trends have also shown opposite direction with forest decreasing and woodland increasing over time.

#### 3.1.1. Accuracy Assessment

Table 2 shows the error matrix with the user's accuracy, producer's accuracy, and kappa coefficient. The results from accuracy assessment revealed that the total accuracy and kappa coefficient were 90.00% and 86.57%, respectively (Table 2), indicating strong agreement between the classified image and the reference data. A value greater than 0.80 (i.e., 80%) represents strong agreement; a value between 0.40 and 0.80 (i.e., 40–80%) represents moderate agreement; and a value below 0.40 (i.e., 40%) represents poor agreement [51]

#### 3.1.2. Land Cover Change Trajectories in the Image Forest Reserve

Over the past three decades, the IFR land cover types have experienced two major trajectories of changes in its land cover (Figure 2). In the first period (1990–2004), large area of woodland changed to forest, and in the second period (2004–2018), large area of forest changed to woodland, shrubland and grassland, and bareland and rock outcrops.

Tables 3 and 4 show the conversion of land cover in the form of a change matrix for the first period (1990–2004) and second period (2004–2018). In the first period (1990–2004), there was a conversion from forest to woodland (1745.19 ha), from woodland to shrubland and grassland (1078.65 ha), and from shrubland and grassland to bareland and rocky outcrops (250.38 ha). In the same period, a change from forest to woodland and from forest to shrub land and grassland was observed though to a small extent.

On the other hand, the second period (2004–2018) showed a further conversion of woodland to forest (1732.50 ha) and from shrubland and grassland to woodland (1076.94 ha). During the same period, another major change from bareland and rocky outcrops to shrubland and grassland (2460.60 ha) was observed. During both periods, very little of forest area was converted to bareland and rocky outcrops in IFR (Table 3).

#### 3.1.3. Gain and Loss of Land Cover in the Image Forest Reserve

**Figure 4:**
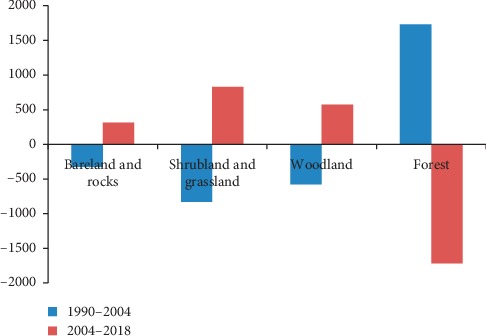
Net change (i.e., gains minus losses) for each land cover class of the IFR for the first period (1990–2004) and the second period (2004–2018).

The net change in the form of gains and losses for each land cover class during the first period (1990–2004) and the second period (2004–2018) is shown in Figure 2. The highest loss was in the forest (1731.96 ha), during the first period, while a significant gain was observed in shrubland and grassland (7,768 km^2^), followed by woodland (577.89 ha). On the other hand, during the second period, the highest loss was observed in bareland and rocky outcrops (2528.10 ha), followed by woodland (572.13 ha) while significant gains were observed in forest (1717.56 ha) and shrubland and grassland (1382.67 ha) (Figure 4).

Grounded on the hectares and percentages of land cover coverage, the land cover types varied between ranges of years (Table 5). ANOVA results for the bareland and rocky outcrops, shrubland and grassland, woodland, and forestland cover types change between 1990 and 2018 showed no significant difference (*P* > 0.05) in land cover change within the land cover types in IFR from 1990 to 2018 (Table 5).

### 3.2. Illegal Human Activities within Image Forest Reserve

The identified human illegal activities in IFR were livestock grazing (Plate 2A), logging for timber which was observed in the forest (*Hagenia abyssinica and Podocarpus latifolius* as the most target trees) (Plate 2B), trespass routes (Plate 2C), wildfires (Plate 2D), and firewood collection (2D), *Brachystegia spiciformis, Ba. Utilis, Julbernardia globiflora, Uapaca kirkiana, Erica mannii, Faure rochetiana*, and *F. saligna* being the most preferred trees. Also, wildlife snaring was evident in IFR (Plate 2F).

#### 3.2.1. Human Illegal Activities Score

The illegal human activities have an influence on land cover types. The human illegal activities' mean score for the affected plots within the land cover types ranged from very low to very high (1–5), while the mean score for all plots within the land cover types ranged from no human activity to low (0 to 2) (Table 6).

The ANOVA test suggested rejection of null hypothesis in a sense that there was a highly significant difference (*P* < 0.05) in the level or rate of human illegal activities within the forest, woodland, shrubland and grassland, and bareland and rocky outcrops in IFR (Table 7).

## 4. Discussion

### 4.1. Land Cover Changes

The land cover types are important for carbon storage and sequestration [52]. The variations in land cover and human interference influence how much biomass and carbon the woody vegetation can hold and indicate a clear need to study these shifting land dynamics [35]. Several studies including Quan et al. [53] have revealed that human needs have led into land cover changes. Plant conservation in the tropical forests faces great challenges that lead to change of the existing vegetation types [54]. Detecting land cover change creates awareness on the root causes of the vegetation cover and plant species alteration Fichera et al. [44, 55–60]. The value of Tanzania's forests is high due to the high potential for royalty collection, exports, and tourism earnings as well as the recycling and fixing of carbon dioxide of globally important biodiversity sites [61]. Land cover types are described based on the existing vegetation types [62, 63]. The findings of this study showed changes in the land cover types in IFR over the three decades (Figure 4). The contraction of forest land cover, while shrub and grassland, and woodland expanded implied that forest cover decreased, allowing the extension of shrub and grassland, and woodland into the formarly forest covered land, even though it did not reveal a net change in the extension of the land cover types along the whole period of 1990 to 2018. Hassan et al. [9] showed that agriculture increase converted forests into farms, and the abandoned former farms became grasslands. Owuabah et al. [64] stated that encroachment of protected areas is evidently damaging the natural vegetation types. Also, Reyers et al. [65] explained that biodiversity hotspot areas face high pressure from great demand for forest resources by the community.

The increase in forest land cover and the decrease in land cover types for the period of 1990–2004 (Table 1) for woodland, bareland and rocky outcrops, shrubland, and grassland show a consistent decrease through different levels, entailing a negative direction of change. This implies that the factors responsible for this trajectory of changes might have not continued over the second period (2004–2018). According to Muhati et al. [66], the decrease in size of an adjascent land cover type gives a room for the extension of the bordering land cover type favoured such as change of woodland is supported by factors favouring the woodlaand, while the change of woodland to forest also is favoured by suitable conditions for forest species to grow. A similar study in China by Zhou et al. [67] , Fichera et al. [60] ,Fichera et al. [79] found that the protected woodland experienced dormant gains and losses, which exemplifies the large dormant category phenomenon, while the agriculture practiced areas and bareland experienced active gains and losses. Many studies have experienced that protected forests are characterized by high plant species diversity playing a great role in the natural ecosystem [68, 69]. It has been highlighted that participatory forest management can sustain protected forests [70] and Lemenih & Bekele [71], even though it has been known that under certain situations, the long-term viability of many participatory forest management projects under JFM agreements in catchment forests seemed questionable, and alternative sources of income and benefits were considered [72, 73], hence leading to land cover change. In general, the slight change in land cover types within the IFR did not cause any significant land cover change of an overall IFR.

### 4.2. Illegal Human Activities as Driver for Land Cover Changes within Image Forest Reserve

**Figure 5:**
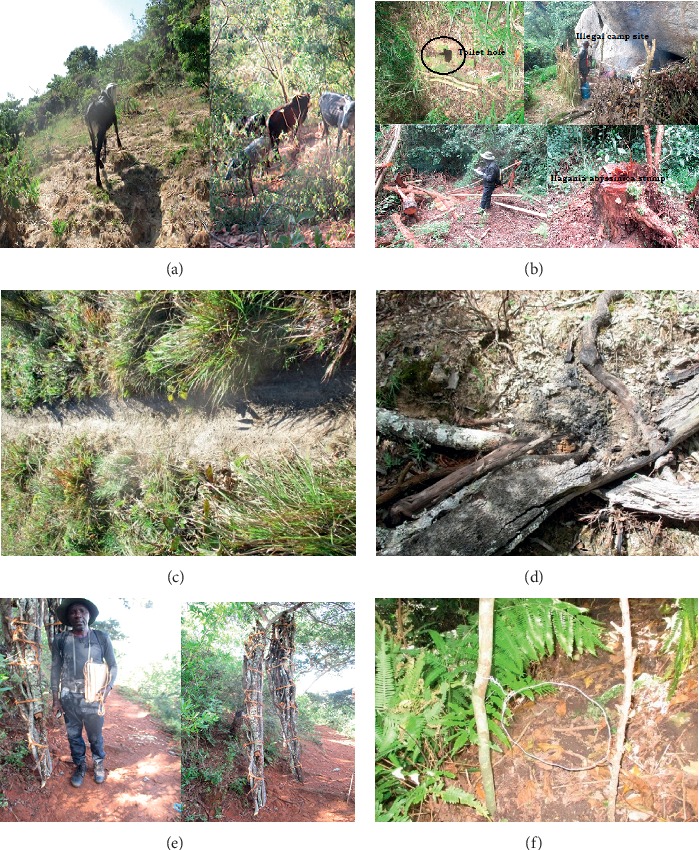
Plate 2. (a) Livestock grazing, (b) logging for timber, (c) illegal paths, (d) wildfires, (e) fire wood collection, and (f) snaring.

The dynamic changes within IFR are accompanied with deforestation, afforestation, and regrowth of forest parts because of natural habitat changes induced by human activities [35] (Figure 5). It has also been stated that diminishing in size of natural vegetation at an alarming rate is mostly accelerated by human exploitation of natural resources, hence leading to vegetation fragmentation [74, 75]. Also, the FAO [76] supported that human rural development strongly depends on natural resources including arable land, timber, and water, a situation which attracts the communities to collect. The URT [77] supported this study's findings that human illegal activities are human unauthorized undertakings conducted in the protected land. The livestock feeding on plants damage both seed bank through trampling impacts, seedlings, saplings, poles and large trees through leeaf damage and debarking. This has been supported by Katan et al. [78] who stated that the vegetation is mostly changed by human activities such as wildfires through conversion of land into agriculture, fresh livestock pastures, and keeping away dangerous wild animals. Logging at IFR, for timber, removes large trees such as *Podocarpus latifolius, Faurea saligna*, and *Hagenia abyssinica*. It has been pinpointed that tree cutting and wildfires open up the tree canopy and hence lead into land cover change [79]. Similar study findings by Muhati et al. [66] supported that the substantial increasing rate of the land use lead to changes in the land cover of Marsabit forest reserve in northern Kenya between 1990 and 2018 and that closed forest hectares had reduced by −38.1% and open forest (−95.4%) with a corresponding increase in hectarage of the agriculture/settlement class (+87.5%).

This, therefore, suggests that the forest loss could be attributing to increased human activities putting pressure on forest resources. The decrease in the closed forest, open forest, and shrubland with a corresponding increase in grassland and bareland suggested an overall deterioration of vegetation cover over the last 27-year period, suggesting land degradation in the natural forest. Butsic et al. [80] discussion supported that as fires threaten human dominated landscapes, fire risk itself has become a driver of landscape change. Trespass routes and snaring (Plate 2F) activities seem to be associated with each other, as routes are illegally created as the local people get into the protected forests while seeking for wild meat. Gray et al. [81] stated that snares are set by the local people whilst engaged in other forest activities, and snares also are used around fields to prevent crop-raiding [81].

Firewood collection involves both collecting the dead and cutting the standing woody materials. Some standing trees are cut and left to dry for some days, the collected firewood from the ground may have an ecological negative effect through damage of nutrient recycling in the ecosystem and damage of micro-organism habitats. Several studies have previously addressed statistically significant positive relationships between fire occurrence and land cover changes and have found that correlation patterns vary for different vegetation types and land-use types [82]. The overall results of the 1 and 2 score implied very low and low human illegal human activities in IFR. This showed a direction that there was a significant difference in the impact of human illegal activities within the land cover types in IFR. Also, the type of human illegal activity depended on the type of forest natural resources the local people needed. In Europe, the vegetation types including the riparian woodlands have been drastically reduced in the surface area along most rivers of the country as a result of human activity along the streams such as agriculture [83]. It has also been estimated that nearly three billion people worldwide primarily depend on forests as their main energy source, thus leading to damage of woody vegetation Crawford and Rodgers [33, 84, 85]. Also, Gumbo et al. [52] supported that wild fires affect vegetation carbon stocks and dynamics in miombo woodlands and that there were studies reporting this effect. IFR faced somewhat random sorts of illegal activities, and some of the parts were affected while others remained intact. The human illegal activities seemed to be based on the type of natural resources needed by the community and the land cover type of its availability. Grazing was abundantly noticeable on woodland followed by bushland, bare rocky outcrops, and forest. Green pastures were common on woodland, while logging was in the forest where the suitable wood resource existed.

## 5. Conclusion and Recommendations

### 5.1. Conclusion

The increase in woodland, shrubland, grassland, bareland, and rocky outcrops means that human activities have impacted negatively the IFR cover. The increase in shrubland and grassland, at the expense of forest lost between 2004 and 2018, suggested inadequte efforts of forest conservation strategies that allowed regular illegal human activities in the protected forest. Through intensified protection measures by the government authority, the illegal human activities may decrease in IFR and the plant species habitats can recover through a stable land cover pattern. Protection can favour a forest cover type to extend through change in woodland and other land cover types. However, the bareland and rocky outcrops can be covered with early succession land cover types such as shrubland and grassland which in the future can be covered by forest patches. The bareland and rocky outcrops apart from being difficult habitats for plants to grow, under no disturbance from goats and cutting of scattered trees, can initiate a succession cycle through the establishment of lower plants such as moss, grasses, ferns, and herbs, finally creating suitable condition for shrubby cover and later the forest trees. The severe human socioeconomic activities have an influence on the land cover change within IFR, and the safety of this forest rests on the intensified conservation efforts on one hand and the alternative means of survival in the local communities.

### 5.2. Recommendations

In order to be able to manage the forest resources of Image Forest Reserve, further research is needed to assess all biological species of image forest reserve, participatory forest management (PFM) is needed to ensure fully participation of the local communities, bylaws and laws should be implemented to minimize illegal damage of forest resources and refurbishment of the forest gaps' restoration and introduce nonwood income-generating projects in IFR such as ecotourism and beekeeping to enhance local people's employment and income, while the forest resources are being protected, and provision of regular education to the community by Tanzania Forest Services including short- and long-term programmes; livestock zero grazing should be encouraged to avoid damage of protected vegetation through browsing and trampling impacts on seed bank, lower plants, and seedlings; awareness creation to alert the community on the need for protection of potential or sustainable utilization for the present generation without jeopardizing the future generation, also forest boundaries, should be made clear to the community to avoid unnecessary encroachment for crop production and settlement.

## Figures and Tables

**Table 1:** Land cover area, change area, and annual rate of change between 1990 and 2018 in the Image Forest Reserve.

	Land cover change						Changed area			
	1990		2004		2018		1990–2004	2004–2018	1990–2004	2004–2018
	Area (ha)	%	Area (ha)	%	Area (ha)	%	Area (ha)	Area (ha)	Area (ha/yr)	Area (ha/yr)
Bareland and rocky outcrops	554.22	6.08	230.67	2.53	544.14	5.97	−323.55	313.47	−23.11	22.39
Scrubland and grassland	1,378.44	15.12	548.01	6.01	1,379.88	15.13	−830.43	831.87	−59.31	59.42
Woodland	3,331.53	36.54	2,753.64	30.20	3,325.95	36.48	−577.89	572.31	−41.27	40.88
Forest	3,853.71	42.27	5,585.67	61.26	3,868.11	42.42	1,731.96	−1717.56	123.71	−122.68
Total	**9,117.90**	**100**	**9,117.9**	**100**	**9,118.08**	**100**				

**Table 2:** Cross-tabulation error matrix of classified versus reference data for 2018.

Classified image	Reference data					
	Bareland and rocky outcrops	Shrubland and grassland	Woodland	Forest	Row total	User's accuracy
Bareland and rocky outcrops	39	1	0	1	41	95.12%
Shrubland and grassland	1	40	1	2	44	90.91%
Woodland	1	2	49	4	56	87.50%
Forest	0	2	5	52	59	88.14%
Column total	41	45	55	59	200	
Producer's accuracy	95.12%	88.89%	89.09%	88.13%		
Total accuracy	**90.00%**					
Kappa statistics	**86.57%**					

**Table 3:** Land cover change matrix between 1990 and 2004 for the IFR.

Land cover	Bareland and rocky outcrops s	shrubland and grassland	Woodland	Forest	Total (2004)	Gain
Bareland and rocky outcrops s	*219.33*	250.38	84.24	0.27	554.22	334.89
Shrubland and grassland	10.44	*271.35*	1078.65	17.91	1378.35	1107.00
Woodland	0.90	26.10	*1558.53*	1745.91	3331.44	1772.91
Forest	0.00	0.00	32.13	*3821.58*	3853.71	32.13
Total (1990)	230.67	547.83	2753.55	5585.67	9117.72	
Loss	11.34	276.48	1195.02	1764.09		

Italic letters indicate that there is no change in the land cover over the time period.

**Table 4:** Land cover change matrix between 2004 and 2018 for the IFR.

Land cover	Bareland and rocky outcrops s	shrubland and grassland	Woodland	Forest	Total (2018)	Gain
Bareland and rocky outcrops s	*219.15*	10.53	0.90	0.00	230.58	11.43
Shrubland and grassland	2460.60	*275.04*	26.73	0.00	2762.37	2487.33
Woodland	78.66	1076.94	*1565.55*	32.40	2753.55	1188.00
Forest	0.27	17.19	1732.50	*3835.71*	5585.67	1749.96
Total (2004)	2758.68	1379.70	3325.68	3868.11		
Loss	2539.53	1104.66	1760.13	32.40		

Italic letters indicate that there is no change in the land cover over the time period.

**Table 5:** Land cover types' change significance test.

ANOVA						
Source of variation	SS	d*f*	MS	*F*	*P* value	F crit
Between groups	0.580716666	2	0.2903583	0.0000000788	1	4.25649
Within groups	33157913.69	9	3684212.63			
Total	33157914.27	11				

**Table 6:** Human illegal activities' score per land cover type in Image Forest Reserve.

Illegal activity	Land cover	Sap	Pa	Tt %ap	Tts	M %ap	M%alp	Ms	Psap	Aps
Wildfire	Woodland	64	2	170		85.00	2.66		5	1
	Shrubland and grassland	28	1	50		25.00	1.79		2	1
	Forest	67	2	195		97.50	2.91		5	1
Sawing pit	Forest	67	2	32.5		16.25	0.49		1	1
Logging	Forest	67	6		24			4.00	1	0
	Woodland	64	7		26			3.71	1	0
	Bareland and rocky outcrops	11	1		1			1.00	1	0
Grazing	Woodland	64	34	2245		66.03	35.08		3	2
	Shrubland and grassland	28	10	340		34.00	12.14		2	1
	Forest	67	12	546		45.50	8.15		3	1
	Bareland and rocky outcrops	11	2	50		25.00	4.55		2	1
Footpath	Forest	67	9	52		5.78	0.78		1	1
Encroachment	Woodland	64	2	100		50.00	1.56		3	1

0 = no illegal activity effect; 1 = very low; 2 = low; 3 = medium; 4 = high; 5 = very high. Sap = sampled affected plots; Pa = plot(s) affected; Tt%ap = total percentage of affected plots; Tts = total number of stumps; M%ap = mean percentage of affected plots; M%/alp = mean percentage of all affected plots; ms = mean score; Psap = percentage of affected plots; Aps = affected plot score.

**Table 7:** Differences in the rate of human illegal activities in Image Forest Reserve.

ANOVA						
Source of variation	SS	d*f*	MS	*F*	*P* value	F crit
Between groups	1183378	9	131486.50	2.49	0.015	2.00
Within groups	4126909	78	52909.10			
Total	**5310288**	**87**				

## Data Availability

The data used to support the findings of this study are available from the corresponding author upon request.

## References

[1] Turner B. L., Clark W. C., Kates R. W., Richards J. F., Mathews J. T., Meyer W. B. (1990). *The Earth as Transformed by Human Action*.

[2] Goudie A. S., Fowler A., Abdellatif E. (2000). *Desertification in the Third Millennium*.

[3] Maikhuri R. K., Nautiyal S., Rao K. S., Gavali R., Saxena K. G., Chandrasekhar K. (2000). Analysis and resolution of protected area-people conflicts in nanda devi biosphere reserve, India. *Environmental Conservation*.

[4] SaxenaGavali S. S., Dhar U., Rawal R. S. (2000). Assessment of fuel resource diversity and utilization patterns in Askot Wildlife Sanctuary in Kumaun Himalaya, India, for conservation and management. *Environmental Conservation*.

[5] Bruner A. G., Gullison R. E., Rice R. E., Fonseca G. A. (2001). Effectiveness of parks in protecting Tropical biodiversity. *Science*.

[6] Trakolis D. (2001). Local people’s perceptions of planning and management issues in Prespes Lakes National Park, Greece. *Journal of Environmental Management*.

[7] Zeng H., Sui D. Z., WU X. B. (2005). Human disturbances on landscapes in protected areas: a case study of the Wolong Nature Reserve. *Ecological Research*.

[8] Mligo C. (2015). Plant species composition and distribution in relation to land use patterns in serengeti ecosystem Tanzania. *Open Journal of Forestry*.

[9] Hassan Z., Shabbir R., Ahmad S. S. (2016). Dynamics of land use and land cover change (LULCC) using geospatial techniques: a case study of Islamabad Pakistan. *Springer Plus*.

[10] Gullison R. E., Frumhoff P. C., Canadell J. G. (2007). ENVIRONMENT: tropical forests and climate policy. *Science*.

[11] Munishi P. K. T., Mringi S., Shirima D. D., Linda S. K. (2010). The role of the miombo woodlands of the southern highlands of Tanzania as carbon sinks. *Journal of Ecology and The Natural Environment*.

[12] Newmark W. D. (1991). Tropical forest fragmentation and the local extinction of understory birds in the eastern Usambara Mountains, Tanzania. *Conservation Biology*.

[13] Lovett J. C., Friis I., van der Maesen L. J. G., van der Burgt X. M., van Medenbach de Rooy J. M. (1996). Some patterns of endemism in the tropical north east and eastern African woody flora. *The Biodiversity of African Plants. Proceedings XIVth AETFAT Congress 22-27 August 1994*.

[14] Moyer D. (1992). *Report on the Natural Resources Consultancy for the Udzungwa Forest Management Plan Project Preparation Mission*.

[15] Lovett J. C., Wasser S. K. (1993). *Biogeography and Ecology of the Rain Forests of Eastern Africa*.

[16] Lovett J. C. (1998). Continuous change in Tanzanian moist forest tree communities with Elevation. *Journal of Tropical Ecology*.

[17] GEF (2002). *Project Brief: Conservation and Management of the Eastern Arc Mountain Forests, Tanzania*.

[18] Myers N., Mittermeier R. A., Mittermeier C. G., da Fonseca G. A. B., Kent J. (2000). Biodiversity hotspots for conservation priorities. *Nature*.

[19] Doody K. Z., Howell K. M., Fanning E., Frontier Tanzania (2001). Frontier Tanzania 2001 new dabaga/ulangambi forest reserve-botanical and forest use report. *Report for the Udzungwa Mountains Forest Management and Biodiversity Conservation Project*.

[20] Brooks T. M., Mittermeier R. A., Mittermeier C. G. (2002). Habitat loss and extinction in the hotspots of biodiversity. *Conservation Biology*.

[21] Lovett J. C. (1998). Importance of the Eastern Arc Mountains for vascular plants. *Journal of East African Natural History*.

[22] Burgess N. D., Nummelin M., Fjeldsa K. M. (1998). Biodiversity and conservation of the eastern arc mountains of Tanzania. *Journal of East African Natural History*.

[23] Burgess N. D., Clarke G. P. (2000). *The Coastal Forests of Eastern Africa*.

[24] WWF-EARPO (2002). *Eastern African Coastal Forest Progamme. Regional Workshop Report. Nairobi*.

[25] WWF-US (2003). Eco-regional reports: eastern arc forest. *Eastern and Southern Africa Biomes*.

[26] Lung T., Schaab G. (2010). A comparative assessment of land cover dynamics of three protected forest areas in tropical eastern Africa. *Environmental Monitoring and Assessment*.

[27] Olson J. M., Maitima J. M. (2006). *Sustainable Intensification of Mixed Crop-Livestock Systems, Land Use Change Impacts and Dynamics (Lucid)*.

[28] Malgorzata B., Johana A., Johannes B., Tuija B. R., Frank J. (2016). Effects of management intensity, function and vegetation on the biodiversity in urban ponds. *Urban Forestry and Urban Greening*.

[29] Grimm N. B., Staudinger M. D., Staudt A. (2013). Climate-change impacts on ecological systems: introduction to a US assessment. *Frontiers in Ecology and the Environment*.

[30] Kimaro J., Lulandala L. (2013). Human influences on tree diversity and composition of a coastal forest ecosystem: the case of ngumburuni forest reserve, rufiji, Tanzania. *International Journal of Forestry Research*.

[31] Mir A. H., Upadhaya K., Choudhury H. (2014). Diversity of endemic and threatened ethno medicinal plant species in Meghalaya, north-east India. *International Research Journal of Environmental Sciences*.

[32] Ayres M. P. (2000). Assessing the consequences of global change for forest disturbance from herbivores and pathogens. *Science of the Total Environment*.

[33] Lombardero R., Sharma P., Browder J. (1992). *Deforestation Problems, Causes and Concerns In Managing of World Forests: Looking for Balance between 71 Conservation and Development*.

[34] Achard F., Eva H. J., Stibig P., Mayaux M. G., Richards J. T., Malingreau J. P. (2002). Determination of deforestation rates of the world’s humid tropical forests. *Science*.

[35] Shirima D. D. (2015). Forest and woodland of Tanzania: interactions between woody Plants Structures, Diversity, carbon stocks and soil nutrient heterogeneity.

[36] Halpern C. B., Spies T. A. (1995). Plant species diversity in natural and managed forests of the Pacific Northwest. *Ecological Applications*.

[37] Spies G., Mangstu T., Gelu Z., Zewdie S. (2013). Forest carbon pools and carbon stock assessment in the context of SFM and REDD*+*. *Awassa University Wondo Genet College of Forestry and Natural Resources Training Manual*.

[38] Vitousek P. M., Mooney H. A., Lubchenco J., Melillo J. M. (1997). Human domination of earth’s ecosystems. *Science*.

[39] UCN (2016). *International Union for Conservation of Nature Union Annual Report*.

[40] Lovett J. C., Congdon C. (1990). Selegu mountain report. *Image Forest Reserve Tanzania. NHS Bull*.

[41] United Republic of Tanzania (2013). Kilolo district council socio-economic profile. The statistics for development. *The Ministry of Finance, National Bureau of Statistics of Tanzania Report*.

[42] Potapov P. A., Yaroshenko S., Turubanova M. (2008). Mapping the world’s intact forest landscapes by remote sensing. *Ecology and Society*.

[43] Douwes E., Rouget M., Diederichs N., Donoghue S. O., Roy K., Roberts D. (2016). The buffels draai Landfill site community reforestation project. *International Journal of Forest Engineering*.

[44] Tottrup C. (2004). Improving tropical forest mapping using multi-date Landsat TM data and pre-classification image smoothing. *International Journal of Remote Sensing*.

[45] Hurtt G. C., Chini L. P., Frolking S. (2019). *Harmonization of land-use scenarios for the period 1500–2100: 600 years of global gridded annual land-use transitions*.

[46] Brown K. A., Parks K. E., Bethell C. A., Johnson S. E., Mulligan M., Kumar L. (2015). Predicting plant diversity patterns in Madagascar: understanding the effects of climate and land cover change in a biodiversity hotspot. *PLoS One*.

[47] Thomson J., Brokawi N., Zimmermanii J. K. (2002). Land use history, environment, and tree composition in a tropical forest. *Ecological Applications*.

[48] Ruffo C. K. (1991). A Report on the Identification of Species for Image Forest Inventory. *Iringa Region*.

[49] Minja T. R. A. (1991). The management and ecology of Tanzania forests. *Image Catchment Forest Reserve Report*.

[50] Miller J. R., Rupert G. (1997). *Beyond ANOVA: Basics of Applied Statistics*.

[51] Jensen J. R. (2004). *Introductory digital image processing: a remote sensing perspective*.

[52] Gumbo D., Clendenning J., Martius C. (2018). How have carbon stocks in central and southern Africa’s miombo woodlands changed over the last 50 years? A systematic map of the evidence. *Environmental Evidence*.

[53] Quan B., Xiao Z., Römkens M. J. M., Bai Y., Lei S. (2013). Spatiotemporal urban land use changes in the changzhutan region of hunan province in China. *Journal of Geographic Information System*.

[54] Maunder M., Clubbe C., Hankamer C., Glove M. (2002). *Plant Conservation in the Tropics. Preservatives and Practice*.

[55] Lillesand T. M., Kiefer R. W., Chipman J. W. (2003). *Remote Sensing and Image Interpretation*.

[56] Tadesse W., Coleman T. L., Tsegaye T. D. Improvement of land use and land cover classification of an urban area using image segmentation from Landsat ETM+ data.

[57] Verburg P. H., Schot P. P., Dijst M. J., Veldkamp A. (2004). Land use change modelling: current practice and research priorities. *Geo Journal*.

[58] Haque M. I., Basak R. (2017). Land cover change detection using GIS and remote sensing techniques: a spatio-temporal study on Tanguar Haor, Sunamganj, Bangladesh. *The Egyptian Journal of Remote Sensing and Space Science*.

[59] Marion K., Ziora M., Dörnhöfer K., Oppelt N., Müller F. (2014). Detecting land use and land cover changes in northern German agricultural landscapes to assess ecosystem service dynamics. *International Association for Landscape Ecology*.

[60] Fichera C. R., Modica G., Pollino M. (2012). Land Cover classification and change-detection analysis using multi-temporal remote sensed imagery and landscape metrics. *European Journal of Remote Sensing*.

[61] United Republic of Tanzania (1998). *National Forest Policy*.

[62] Handa C., Alvarez M., Becker M. (2012). Opportunistic vascular plant introduction in agricultural wetlands of East Africa. *International Journal of AgriScience*.

[63] Bryn A., Strand G.-H., Angeloff M., Rekdal Y. (2018). Land cover in Norway based on an area frame survey of vegetation types. *Norsk Geografisk Tidsskrift-Norwegian Journal of Geography*.

[64] Owubah C. E., Donkor N. T., Nsenkyire R. D. (2000). Forest reserve encroachment: the case of tano-ehuro forest reserve in western Ghana. *The International Forestry Review*.

[65] Reyers B. O., Farrell P. J., Cowling R. M., Egoh B. N., Le Maitre D. C., Vlok J. H. (2009). Ecosystem services, land-cover change, and stakeholders: finding a sustainable foothold for a semiarid biodiversity hotspot. *Ecology and Society*.

[66] Muhati G. L., Olago D., Olaka L. (2018). Land use and land cover changes in a sub-humid montane forest in an arid setting: a case study of the Marsabit forest reserve in northern Kenya. *Global Ecology and Conservation*.

[67] Zhou P., Huang J., Pontius R., Hong H. (2014). Land classification and change intensity analysis in a coastal watershed of southeast China. *Sensors*.

[68] Hackenberg J., Wassenberg M., Spiecker H., Sun D. (2015). Non-destructive method for biomass prediction combining TLS derived tree volume and wood density. *Forests*.

[69] Tamungang S. A., Onabid M. A., Awa T., Balinga V. (2016). Habitat preferences of the grey parrot in heterogeneous vegetation landscapes and their conservation implications. *International Journal of Biodiversity*.

[70] United Republic of Tanzania (2008). Participatory forest management in Tanzania: booklet of facts and figures. *Minisitry of Natural Resources and Tourism*.

[71] Lemenih M., Bekele M. (2008). Participatory forest management: best practices, lesson learnt and challenges encountered. *The Ethiopian and Tanzanian Experiences Report*.

[72] DANIDA, Environment, Peace and Stability Facility (MIFRESTA) Environment Support Programme (ESP), Participatory Forest Management (2003–2007), Participatory Forest Management in Tanzania Component of the Environmental Support Programme Tanzania Component Document. Ref. No. 104. Tanzania.1.MFS.0, 2002

[73] Bomley T., Idd S. (2009). Participatory forest management in Tanzania: 2003–2009. *Lessons Learned and Experiences to Date Report*.

[74] Dupuy D. J., Labat J. N., Robovohitra R., Villers J. F., Bosser J., Moat J. (2002). *Ecology of the Leguminosae in Madagascar*.

[75] Mugume S., Isabirye-Basuta G., Otali E., Reyna-Hurtado C. A. (2015). How do human activities influence the status and distribution of terrestrial mammals in forest reserves?. *Journal of Mammalogy*.

[76] FAO (1985). *Gude to Extension Training*.

[77] United Republic of Tanzania (2007). *The wildlife policy of Tanzania*.

[78] Katan J. Z., Mawinda S., Mugasha W. A. (2019). *Understanding Plantation and Natural Forests: A Hand Book for Forest Practioners*.

[79] Abella S. R., Springer J. D. (2015). Effects of tree cutting and fire on understory vegetation in mixed conifer forests. *Forest Ecology and Management*.

[80] Butsic V., Kelly M., Moritz M. A. (2015). Land use and wildfire: a review of local interactions and tele-connections. *Land*.

[81] Gray T. N. S., Hughes A. C., Laurence W. F (2018). The wildlife snaring crisis: an insidious and pervasive threat to biodiversity in Southeast Asia. *Biodiversity and Conservation*.

[82] Hugh E., Lambin E. F. (2000). Fires and land-cover change in the tropics: a remote Sensing analysis at the landscape scales. *Journal of Biogeography*.

[83] Naiman R. J., Décamps H. (2015). *Disturbance and Agents of Change. Riparia*.

[84] LikensMcClain A., Kewessa G. (2015). Woody species diversity in traditional agroforestry practices of dellomenna district, southeastern Ethiopia: implication for maintaining native woody species. *International Journal of Biodiversity*.

[85] Crawford K. M., Rodgers J. A. (2012). Plant species diversity and genetic diversity within a dominant species interactively affect plant community biomass. *Journal of Ecology*.

